# What predicts university students’ behavioral intention to use education influencers in higher education? A study through the lens of the UTAUT model and self-determination theory

**DOI:** 10.3389/fpsyg.2026.1784297

**Published:** 2026-08-05

**Authors:** You Zhou, Xin Tang

**Affiliations:** 1 School of Education, Jiangsu Open University, Nanjing, China; 2 School of Mathematics and Statistics, Hunan Normal University, Changsha, China

**Keywords:** education influencers, higher education, PLS-SEM, self-determination theory, UTAUT

## Abstract

This study investigates the factors influencing university students’ behavioral intentions to use education influencers by integrating the Unified Theory of Acceptance and Use of Technology (UTAUT) and Self-Determination Theory (SDT). A quantitative survey was conducted with 335 university students in China, and the collected data were analyzed using partial least squares structural equation modeling (PLS-SEM) with SmartPLS 4. The findings showed that performance expectancy, social influence, and self-determination significantly influenced behavioral intention. In addition, effort expectancy, social influence, and facilitating conditions significantly influenced self-determination. However, effort expectancy and facilitating conditions did not directly affect behavioral intention. The results further indicated that self-determination contributed to explaining the motivational mechanisms underlying university students’ intentions to use education influencers. Overall, the study suggests that both technology-related perceptions and motivational mechanisms are important in understanding students’ engagement with education influencers in digital learning environments. The findings provide practical implications for educators, higher education institutions, and education influencers seeking to support university students’ learning experiences through social media-based educational content.

## 1 Introduction

Students’ learning experiences have been increasingly shaped by the rapid integration of social media into educational environments (Jukes et al., 2010; Feito and Brown, 2018). In recent years, social media platforms such as YouTube, TikTok, Instagram, and Bilibili have evolved beyond entertainment and communication tools and become important spaces for informal learning and knowledge sharing. Within these environments, education influencers have emerged as a new form of digital learning facilitator. Education influencers are social media content creators who share educational knowledge, learning strategies, academic experiences, and professional advice with online audiences through videos, livestreams, and interactive online content (Freberg et al., 2011; Kukulska-Hulme et al., 2022).

Compared with traditional educational resources, education influencers often provide more flexible, accessible, and interactive learning experiences. Through social media platforms, university students can access educational content at any time, communicate with influencers and peers, and participate in online learning communities. Previous studies have suggested that social media-based learning environments may enhance students’ engagement, participation, and collaborative learning experiences (Blayone et al., 2017). In addition, education influencers may help students better understand complex concepts by presenting educational content in more practical, personalized, and visually engaging ways. For example, influencers may explain difficult academic concepts through real-world applications, share effective learning strategies, or provide guidance related to academic development and career planning.

The growing popularity of education influencers is particularly relevant in higher education contexts. University students increasingly rely on digital platforms to obtain information, develop learning strategies, and supplement formal classroom learning. In information-rich digital environments, students are often required to filter large amounts of educational content and identify useful learning resources efficiently (Zhong, 2022). In this context, education influencers may play an increasingly important role in supporting students’ self-directed learning experiences, academic engagement, and access to educational resources.

Although education influencers are becoming increasingly important in digital learning environments, existing studies have mainly focused on social media usage, online learning platforms, or general educational technologies rather than education influencers specifically (Blayone et al., 2017; Alowayr and Al-Azawei, 2021; Zacharis and Nikolopoulou, 2022). Comparatively limited attention has been given to university students’ behavioral intentions toward using education influencers as learning resources. Moreover, prior studies have primarily examined technology acceptance from cognitive or technological perspectives, while the motivational and psychological mechanisms underlying students’ engagement with education influencers remain underexplored. Understanding university students’ behavioral intentions toward education influencers is important because education influencers increasingly shape how students access knowledge, develop learning strategies, and engage with digital learning communities. As social media-based learning becomes more widespread in higher education, identifying the factors that influence students’ intentions may help educators and institutions better support students’ learning experiences in digital environments. The present study contributes to the literature in several ways. First, it extends existing research on educational technology adoption by focusing specifically on education influencers in higher education contexts. Second, the study integrates the Unified Theory of Acceptance and Use of Technology (UTAUT) and Self-Determination Theory (SDT) to provide a more comprehensive explanation of university students’ behavioral intentions. Third, unlike prior studies that mainly focused on direct technology acceptance relationships, this study examines both the direct and indirect pathways through which UTAUT-related perceptions influence behavioral intention through motivational mechanisms associated with self-determination. Accordingly, this study addresses the following research questions:

*RQ1*: What factors influence university students’ intentions to use education influencers?

*RQ2*: What role does self-determination play in university students’ behavioral intentions to use education influencers?

The remainder of this paper is organized as follows. Section 2 reviews the relevant literature and develops the research hypotheses. Section 3 describes the methodology, including the research design, measurement instrument, data collection procedures, and data analysis techniques. Section 4 presents the results of the PLS-SEM analysis. Section 5 discusses the findings, implications, limitations, and future research directions. Finally, section 6 concludes the study.

## 2 Literature review

### 2.1 Education influencers

Education influencers are social media content creators who attract large audiences by sharing educational knowledge, learning experiences, and academic perspectives through engaging digital content. As a specific kind of social media influencers, education influencers utilize social media platforms to deliver educational content, provide insights, and talk with their viewers via videos, images, and infographics (Gil-Quintana et al., 2020; Kukulska-Hulme et al., 2022). With websites like YouTube, Twitter, Instagram, Pinterest, and TeachersPayTeachers appearing as essential for educational content (Fyfield et al., 2021; Shapiro et al., 2019; Staudt Willet, 2019), these bloggers are influential in both professional and personal educational contexts. Online educational creators such as Khan Academy have become important sources of learning content for many students worldwide (Fang et al., 2024). Education influencers help their peers navigate their lives by providing academic guidance and emotional support through social media (Raaper et al., 2024). Hirst (2022) investigates how education influencers from renowned companies like Oxford and Cambridge normalize the higher-level educational experience and encourage non-regular students to apply. These influencers collaborate to offer their followers both academic and emotional support (Hirst, 2022).

The rise of education influencers aligns with broader trends in modern education, highlighting flexibility and freedom. Innovative learning environments are promoted using the appeal of education influencers (Kukulska-Hulme et al., 2022). Education influencers’ efforts to create accessible, real-time learning prospects have a huge impact on countless teachers worldwide. Education influencers usually possess tremendous knowledge and experience. According to their in-depth knowledge of the field of education or their own experiences (Chen et al., 2025), they can offer university students recommendations for topics like choosing major directions and course arrangements (Chen et al., 2025). They offer valuable advice on managing and checking stress, successful research techniques, and time management techniques, which can help college students increase their grades and study efficiency. They enable students to clearly define their career goals and give university students a better understanding of the characteristics and requirements of various career paths (Qiyang and Jung, 2019). University individuals may experience a variety of pressures and challenges throughout their academic and professional lives. Education influencers may promote methods and experience in dealing with tension, anxiety, and sadness, aiding people to maintain a good emotional state. Through social media platforms, education influencers can provide a space for communication and interaction for university students, helping them make like-minded friends, expand their social networks, and enhance their sense of belonging (Donhauser and Beck, 2021). They recommend high-quality learning resources such as online courses, study websites, books, and academic journals, helping university students enrich their knowledge reserves and broaden their learning horizons. By explaining complex knowledge and concepts in a lively and interesting way, they can arouse university students’ interest in learning and make learning more attractive.

### 2.2 UTAUT

Davis introduced the first-generation technology acceptance model (TAM) in 1989 (Davis et al., 1989), which identified two primary cognitive beliefs influencing users’ acceptance of technology: perceived usefulness and perceived ease of use. Venkatesh and Davis’ second-generation technology acceptance model (TAM2) examined how social and cognitive processes affect specific technologies’ perceived usefulness, behavioral intention, and usage behavior (Venkatesh and Davis, 2000). Venkatesh et al. (2003) incorporated the concepts of technological knowledge, organizational behavior, social cognitive theory, and other relevant areas. According to the model, user acceptance and use of technology were influenced primarily by performance expectancy (PE), effort expectancy (EE), social influence (SI), and facilitating conditions (FC). However, four moderating factors related to individual differences were identified: gender, age, experience, and voluntariness of use. The degree to which people believe using the software will enhance their work efficiency is known as performance expectancy. Effort expectancy refers to the degree of ease associated with the use of the system. Social influence refers to the extent to which individuals perceive that important others believe they should use the system. Facilitating conditions refer to users’ belief in the availability of a well-organized, skilled infrastructure and resources to support their system usage. As per the UTAUT model (Venkatesh et al., 2003), performance expectancy, effort expectancy, and social influence directly influence users’ behavioral intention. Moreover, facilitating conditions directly affect usage behavior, and users’ behavioral intention directly influences usage behavior. The UTAUT model is widely used because of its nearly 70% explanatory power of user behavior, which is significantly higher than that of other technology acceptance models (Al-Nuaimi and Al-Emran, 2021; Alghazi et al., 2020; Aytekin et al., 2022; Granić, 2022; Qiao et al., 2021; Shaqrah and Almrs, 2022). Since the UTAUT model identifies the primary factors influencing technology usage behavior, it can be used as a tool to predict university students’ behavioral intention regarding their use of new technologies. At present, many extended versions of the UTAUT model are being developed to enhance its explanatory capacity (Ahmed et al., 2022; Ahmed et al., 2024a; Hermita et al., 2023). Accordingly, the present study employs a theoretical framework based on UTAUT to explore the factors that shape university students’ behavioral intentions to use education influencers. Although UTAUT2 extends the original UTAUT model by incorporating additional constructs such as hedonic motivation, price value, and habit (Venkatesh et al., 2012), the present study adopted the original UTAUT framework for several reasons. First, UTAUT2 was primarily developed for consumer technology adoption contexts, whereas this study focuses on educational technology-related behavioral intention in higher education environments. Second, the present study integrates Self-Determination Theory (SDT) to explain motivational and psychological mechanisms underlying behavioral intention. Some motivational dimensions introduced in UTAUT2 overlap conceptually with SDT-related constructs. Therefore, adopting the original UTAUT framework together with SDT was considered more theoretically appropriate for the objectives of this study.

### 2.3 Self-determination theory

The self-determination theory (SDT), a comprehensive framework for understanding human motivation, has been effectively utilized in diverse fields including parenting, education, health care, sports, physical activity, and psychotherapy (Deci and Ryan, 1985; Ryan and Deci, 2017). Consisting of six interrelated mini-theories, SDT builds upon its established knowledge framework to provide a more nuanced understanding of human motivation (Ryan and Deci, 2019; Vansteenkiste et al., 2010). According to SDT, individuals possess an inherent drive for psychological growth, skill acquisition, and forming interpersonal connections. Nevertheless, these natural tendencies do not emerge automatically; they need supportive environments to flourish (Ryan and Deci, 2020). SDT emphasizes the necessity of supporting three basic psychological needs (autonomy, competence, and relatedness) to promote healthy development (Ryan and Deci, 2020). Within SDT, the assessment of educational environments mainly focuses on determining the extent to which these fundamental needs are met. Autonomy involves an individual’s sense of control and ownership over their actions, including the freedom to make choices and the experience of self-determination. Competence relates to a sense of mastery and the belief in one’s ability to achieve success and progress, which is characterized by the desire to effectively engage with one’s environment. Lastly, relatedness is marked by the need to feel a sense of belonging and to establish meaningful, secure emotional bonds with peers and others (Ryan and Deci, 2017; Ryan and Deci, 2020).

Although UTAUT has been widely applied to explain technology acceptance and behavioral intention in educational contexts, the model mainly emphasizes cognitive and system-related determinants, such as performance expectancy, effort expectancy, social influence, and facilitating conditions. However, the use of education influencers is not solely a technology acceptance behavior, but also a self-directed and motivational learning behavior occurring within social media environments. Therefore, relying exclusively on UTAUT may not fully explain the underlying psychological mechanisms that motivate university students to engage with education influencers. Self-Determination Theory complements UTAUT by emphasizing individuals’ intrinsic motivation and the satisfaction of three basic psychological needs: autonomy, competence, and relatedness. In the context of education influencers, university students often voluntarily choose educational content based on their personal interests, perceived competence, and sense of social connection with online communities. These characteristics align closely with the core assumptions of SDT.

**FIGURE 1:**
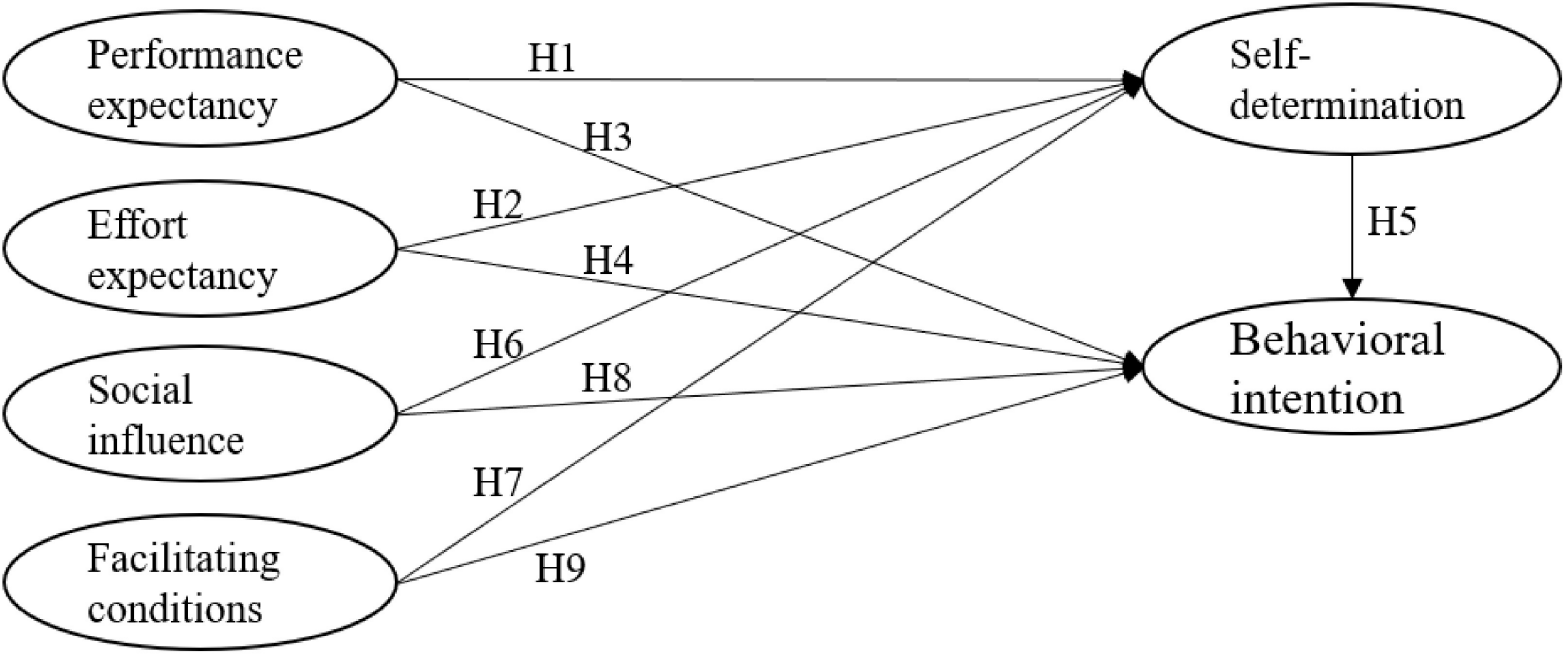
A conceptual model of usage of education influencers for university students.

Existing studies integrating UTAUT and SDT have primarily focused on the indirect influence of technology acceptance factors on behavioral intention through motivational mechanisms. However, limited attention has been given to whether UTAUT constructs may simultaneously exert both direct and indirect effects on behavioral intention when SDT is incorporated into the model. In the context of education influencers, this distinction is particularly important because students’ intentions may be shaped not only by cognitive evaluations of technology use, but also by intrinsic motivational processes. Therefore, the present study retains UTAUT as the primary explanatory framework while incorporating SDT as a mediating mechanism to examine both the direct and indirect pathways influencing behavioral intention. The proposed conceptual model is presented in Figure 1.

*H1:* Performance expectancy affects university students’ self-determination when using education influencers.

*H2:* Effort expectancy affects university students’ self-determination when using education influencers.

*H3:* Performance expectancy affects university students’ behavioral intention to use education influencers.

*H4:* Effort expectancy affects university students’ behavioral intention to use education influencers.

*H5:* Self-determination affects university students’ behavioral intention to use education influencers.

*H6:* Social influence affects university students’ self-determination when using education influencers.

*H7:* Facilitating conditions affect university students’ self-determination when using education influencers.

*H8:* Social influence affects university students’ behavioral intention to use education influencers.

*H9:* Facilitating conditions affect university students’ behavioral intention to use education influencers.

## 3 Methodology

### 3.1 Research design

This study adopted a quantitative research design to investigate the factors influencing university students’ behavioral intentions toward using education influencers. Based on the integrated UTAUT-SDT framework, a cross-sectional survey approach was employed to collect data from university students. The proposed hypotheses were tested using partial least squares structural equation modeling (PLS-SEM).

### 3.2 Measures and instrument development

The questionnaire was developed based on the Unified Theory of Acceptance and Use of Technology (UTAUT) and Self-Determination Theory (SDT). Questionnaire items were adapted from previously validated scales related to educational technology adoption and motivational learning research. Specifically, the items measuring performance expectancy, effort expectancy, social influence, facilitating conditions, and behavioral intention were adapted from prior UTAUT-based studies (e.g., Alowayr and Al-Azawei, 2021; Venkatesh et al., 2003), while the self-determination items were developed based on the dimensions of autonomy, competence, and relatedness derived from SDT literature (Ryan and Deci, 2020).

Because the target participants were Chinese university students, the questionnaire was prepared in Chinese. The questionnaire items were carefully reviewed and refined to ensure clarity, readability, and contextual appropriateness. Prior to the formal survey, a pilot study involving university students was conducted on February 5, 2025, to evaluate the clarity and reliability of the questionnaire items. Based on participant feedback and preliminary reliability analysis, several wording adjustments were made to improve comprehensibility and consistency. Following the pilot study, a total of twenty-three items were finalized for the formal survey.

The questionnaire consisted of two sections. The first section collected participants’ demographic information, including gender and academic degree level (bachelor’s or master’s degree). The second section measured the six latent constructs included in the conceptual model: performance expectancy, effort expectancy, social influence, facilitating conditions, self-determination, and behavioral intention. All items were measured using a 5-point Likert-type scale ranging from 1 (“strongly disagree”) to 5 (“strongly agree”). The detailed measurement items used for each construct are presented in Appendix 1.

### 3.3 Data collection procedure

The research was conducted in Nanjing. To distribute the questionnaire, the Questionnaire Star website^1^ was employed. With the help of several university teachers, the questionnaire link was shared with their students via WeChat on February 21, 2025. Before completing the questionnaire, participants were informed of the study purpose, the voluntary nature of participation, their right to withdraw at any time without penalty, and the measures taken to protect confidentiality, anonymity, and data security. No personally identifiable information was collected. Responses completed in under 2 min were excluded because this duration was considered insufficient for participants to read and answer all 23 questionnaire items attentively. Based on this criterion, 18 responses were removed during the data screening process. After data cleaning, the final sample consisted of 335 university students aged between 18 and 25 who had experience using education influencers. Table 1 provides details of the demographic characteristics of the respondents.

**TABLE 1:** Demographic data of the respondents.

Demographic	Type	Number (*N* = 335)	Percentage (%)
Gender	Male	221	66.0
	Female	114	34.0
Degree	Bachelor	216	64.5
	Master	119	35.5

### 3.4 Data analysis

SPSS version 26 was used to conduct data cleaning and descriptive statistical analysis. The Kolmogorov-Smirnov test confirmed that the data were not normally distributed (Drezner et al., 2010). Therefore, partial least squares structural equation modeling (PLS-SEM) using SmartPLS 4 was employed to test the proposed hypotheses. Compared with covariance-based structural equation modeling (CB-SEM), PLS-SEM is considered more appropriate for prediction-oriented studies, complex models, and data with non-normal distributions (Ahmed et al., 2024b; Hair et al., 2022). The PLS-SEM analysis was conducted in two stages: measurement model evaluation and structural model evaluation. The measurement model assessment included indicator reliability, internal consistency reliability, convergent validity, and discriminant validity. Specifically, factor loadings, Cronbach’s alpha, composite reliability (CR), average variance extracted (AVE), the Fornell-Larcker criterion, and the heterotrait-monotrait ratio (HTMT) were evaluated. The structural model assessment included collinearity diagnostics, path coefficient analysis, coefficient of determination (R^2^), effect size (f^2^), and model fit indices. The results were generated using the PLS algorithm and bootstrapping procedures in SmartPLS 4.

## 4 Results

The analysis proceeded in two stages. First, the reflective measurement model was evaluated in terms of indicator reliability, internal consistency reliability, convergent validity, and discriminant validity. Second, the structural model was assessed using collinearity diagnostics, path coefficients, *R*^2^, f^2^, Q^2^, and model fit indices.

### 4.1 Measurement model evaluation

The reflective measurement model’s reliability and validity were assessed. According to Hair et al. (2019), indicators with outer loadings above 0.708 are considered desirable. All indicator outer loadings ranged from 0.757 to 0.947, exceeding the recommended threshold of 0.708 (Hair et al., 2019), thereby indicating satisfactory indicator reliability. All *t*-values exceeded the recommended threshold of 1.96 at the 0.05 significance level (Hair et al., 2019). The results demonstrate that all items have satisfactory indicator reliability. Subsequently, Cronbach’s alpha (α), the exact reliability coefficient (ρ_*A*_), and composite reliability (CR) (ρ_*C*_) were calculated to assess the internal consistency and reliability of each construct, ideally ranging from 0.70 to 0.95. Cronbach’s alpha, rho_A, and composite reliability (CR) values ranged from 0.905 to 0.937, 0.907 to 0.937, and 0.934 to 0.959, respectively, all exceeding the recommended threshold of 0.70 (Hair et al., 2019). Consequently, all constructs show good internal consistency reliability (Hair et al., 2019). In terms of convergent validity, the average variance extracted (AVE) values should exceed 0.5. The AVE values ranged from 0.739 to 0.887, all above the recommended threshold of 0.50, indicating satisfactory convergent validity (Hair et al., 2019). Table 2 contains more details.

**TABLE 2:** Results of reliability and convergent validity test.

Constructs	Indicators	Outer loadings	*t*-values	Cronbach’s alpha (α)	Exact reliability coefficient (ρ_A_)	Composite reliability (ρ_C_)	Average variance extracted (AVE)
Performance expectancy (PE)	PE1	0.940	59.173	0.937	0.937	0.959	0.887
	PE2	0.947	61.001				
	PE4	0.939	78.330				
Effort expectancy (EE)	EE1	0.915	73.270	0.936	0.936	0.954	0.838
	EE2	0.922	62.505				
	EE3	0.912	52.247				
	EE4	0.912	71.394				
Social influence (SI)	SI1	0.867	36.928	0.905	0.907	0.934	0.779
	SI2	0.878	51.897				
	SI3	0.893	56.403				
	SI4	0.892	43.900				
Facilitating conditions (FC)	FC1	0.924	86.800	0.921	0.922	0.950	0.864
	FC3	0.937	98.304				
	FC4	0.928	77.815				
Self-determination (SD)	AT1	0.757	29.730	0.929	0.936	0.944	0.739
	AT2	0.914	85.376				
	CP1	0.909	77.223				
	CP2	0.894	60.094				
	RN1	0.830	39.152				
	RN2	0.842	38.561				
Behavioral intention (BI)	BI1	0.924	67.256	0.927	0.927	0.953	0.872
	BI2	0.933	71.413				
	BI3	0.944	72.045				

Moreover, this study also examined the degree of differentiation among individual constructs (Henseler et al., 2016). For discriminant validity to be confirmed, the diagonal elements, which are the square roots of the average variance extracted (AVE), for each latent variable need to be greater than the variable’s highest correlation. Table 3 illustrates that the bolded square roots of the AVEs for each construct, on the diagonal, are higher than the correlation coefficients, thus satisfying the required conditions.

**TABLE 3:** Results of the Fornell-Larcker test for assessing discriminant validity.

Constructs	BI	PE	EE	SI	FC	SD
Behavioral intention (BI)	**0.934**	**0.942**	**0.915**	**0.883**	**0.929**	**0.860**
Performance expectancy (PE)	0.805					
Effort expectancy (EE)	0.701	0.729				
Social influence (SI)	0.809	0.776	0.770			
Facilitating conditions (FC)	0.539	0.507	0.730	0.672		
Self-determination (SD)	0.627	0.556	0.662	0.655	0.659	

Bold diagonal values represent the square roots of the average variance extracted (AVE).

The distinctiveness of each construct within the model was established by the study through the application of a stringent Heterotrait-Monotrait Ratio (HTMT) criterion. Table 4 shows that all constructs in the model had HTMT values below 0.90 (Henseler et al., 2015). If the HTMT value is above 0.90, it suggests insufficient discriminant validity (Hair et al., 2019; Hair et al., 2022). With the highest HTMT value at 0.881 (Table 4), the threshold of 0.90 was not exceeded, thus affirming the discriminant validity of the measurement model (Hair et al., 2018). Thus, the evaluation results demonstrate that discriminant validity has been achieved.

**TABLE 4:** Results of the HTMT test for assessing discriminant validity.

Constructs	BI	PE	EE	SI	FC	SD
Effort expectancy (EE)	0.752	0.777	0.836	0.735	0.712
Social influence (SI)	0.881	0.842			
Facilitating conditions (FC)	0.583	0.544	0.786		
Self-determination (SD)	0.653	0.576	0.701	0.697	

### 4.2 Structural model evaluation

Once the reliability and validity of the model were confirmed, the PLS results of the structural model were examined to investigate the relationships among the constructs. The evaluation covered various metrics, including collinearity, path connectivity, and the model’s ability to explain variance (Hair et al., 2019). Nevertheless, Henseler et al. (2016) emphasized that the overall model fit should be assessed before the evaluation. This principle is especially relevant for the reflective measurement model (Henseler et al., 2016). In this study, the model fit was determined by calculating the standardized root mean square residual (SRMR) and normed fit index (NFI). A model is deemed well-fitted when the SRMR is below 0.08. Moreover, an NFI value above 0.80 is considered acceptable (Henseler et al., 2016; Ziggers and Henseler, 2016). Given an SRMR of 0.067 and an NFI of 0.867, the conceptual model for this study is considered appropriate. Inner VIF values were examined to assess collinearity among the predictor constructs in the structural model. All values were below 5, indicating that collinearity was not at a critical level, although several values exceeded the more conservative threshold of 3 (Kock, 2015). Ideally, the VIF value should be below 3 and, at the very least, not go beyond 5 (Hair et al., 2019). Table 5 shows that all VIF values are under 5, indicating that multicollinearity is not a concern in this research.

**TABLE 5:** Results of evaluating the collinearity problem (inner model’s VIF values).

Constructs	BI	PE	EE	SI	FC	SD
Behavioral intention (BI)						
Performance expectancy (PE)	2.956					2.946
Effort expectancy (EE)	3.685					3.604
Social influence (SI)	3.734					3.614
Facilitating conditions (FC)	2.619					2.392
Self-determination (SD)	2.148					

Direct effects, specific indirect effects, and total effects were estimated using 5,000 bootstrap samples. As shown in Table 6, hypotheses H2, H3, H5, H6, H7, and H8 were supported, whereas hypotheses H1, H4, and H9 were not supported. Specifically, effort expectancy (β = 0.194, *p* < 0.05), social influence (β = 0.236, *p* < 0.05), and facilitating conditions (β = 0.325, *p* < 0.001) have statistically significant positive effects on self-determination. Performance expectancy, social influence, and self-determination significantly influenced behavioral intention, whereas effort expectancy and facilitating conditions did not show significant direct effects. The specific indirect effects and total effects are presented in Tables 7, 8, respectively.

**TABLE 6:** Significance testing results of the structural model path coefficients.

Relationships	Path coefficients (β)	*t*-values	*p*-values	95% CI	Significance (*p* < 0.05)
H1: performance expectancy → self-determination	0.066	0.947	0.344	[−0.072, 0.205]	No
H2: effort expectancy → self-determination	0.194	2.621	0.009	[0.043, 0.339]	Yes
H3: performance expectancy → behavioral intention	0.414	7.677	*p* < 0.001	[0.306, 0.518]	Yes
H4: effort expectancy → behavioral intention	0.037	0.595	0.552	[−0.084, 0.155]	No
H5: Self-determination → behavioral intention	0.153	2.664	0.008	[0.048, 0.270]	Yes
H6: social influence→ self-determination	0.236	2.969	0.003	[0.072, 0.384]	Yes
H7: facilitating conditions→ self-determination	0.325	4.618	*p* < 0.001	[0.182, 0.455]	Yes
H8: social influence→ behavioral intention	0.407	5.935	*p* < 0.001	[0.271, 0.538]	Yes
H9: facilitating conditions→ behavioral intention	−0.072	1.276	0.202	[-0.186, 0.035]	No

**TABLE 7:** Significance analysis of the indirect effects.

Relationships	Total indirect effects	Indirect effects (β)	*T*-values	*P-*values	Significance (*p* < 0.05)
Performance expectancy→ self-determination →behavioral intention	0.010	0.010	0.851	0.395	No
Effort expectancy→ self-determination → Behavioral intention	0.030	0.030	1.809	0.071	No
Social influence→ self-determination →Behavioral intention	0.036	0.036	1.875	0.061	No
Facilitating conditions → self-determination →behavioral intention	0.050	0.050	2.289	0.022	Yes

The significance of indirect effects was assessed based on two-tailed p-values, with *p* < 0.05 indicating statistical significance.

**TABLE 8:** Significance testing results of the total effects.

Relationships	Total effects (β)	*T-*values	*P-*values	95% CI	Significance (*p* < 0.05)
Performance expectancy → Self-determination	0.066	0.947	0.344	[–0.072, 0.205]	No
Effort expectancy → Self-determination	0.194	2.621	0.009	[0.043, 0.339]	Yes
Performance expectancy → Behavioral intention	0.425	7.878	*p* < 0.001	[0.316, 0.527]	Yes
Effort expectancy → Behavioral intention	0.067	1.105	0.269	[–0.054, 0.180]	No
Self-determination → Behavioral intention	0.153	2.664	0.008	[0.048, 0.270]	Yes
Social influence → Self-determination	0.236	2.969	0.003	[0.072, 0.384]	Yes
Facilitating conditions → Self-determination	0.325	4.618	*p* < 0.001	[0.182, 0.455]	Yes
Social influence → Behavioral intention	0.443	6.589	*p* < 0.001	[0.309, 0.574]	Yes
Facilitating conditions → Behavioral intention	–0.023	0.433	0.665	[–0.126, 0.078]	No

**FIGURE 2:**
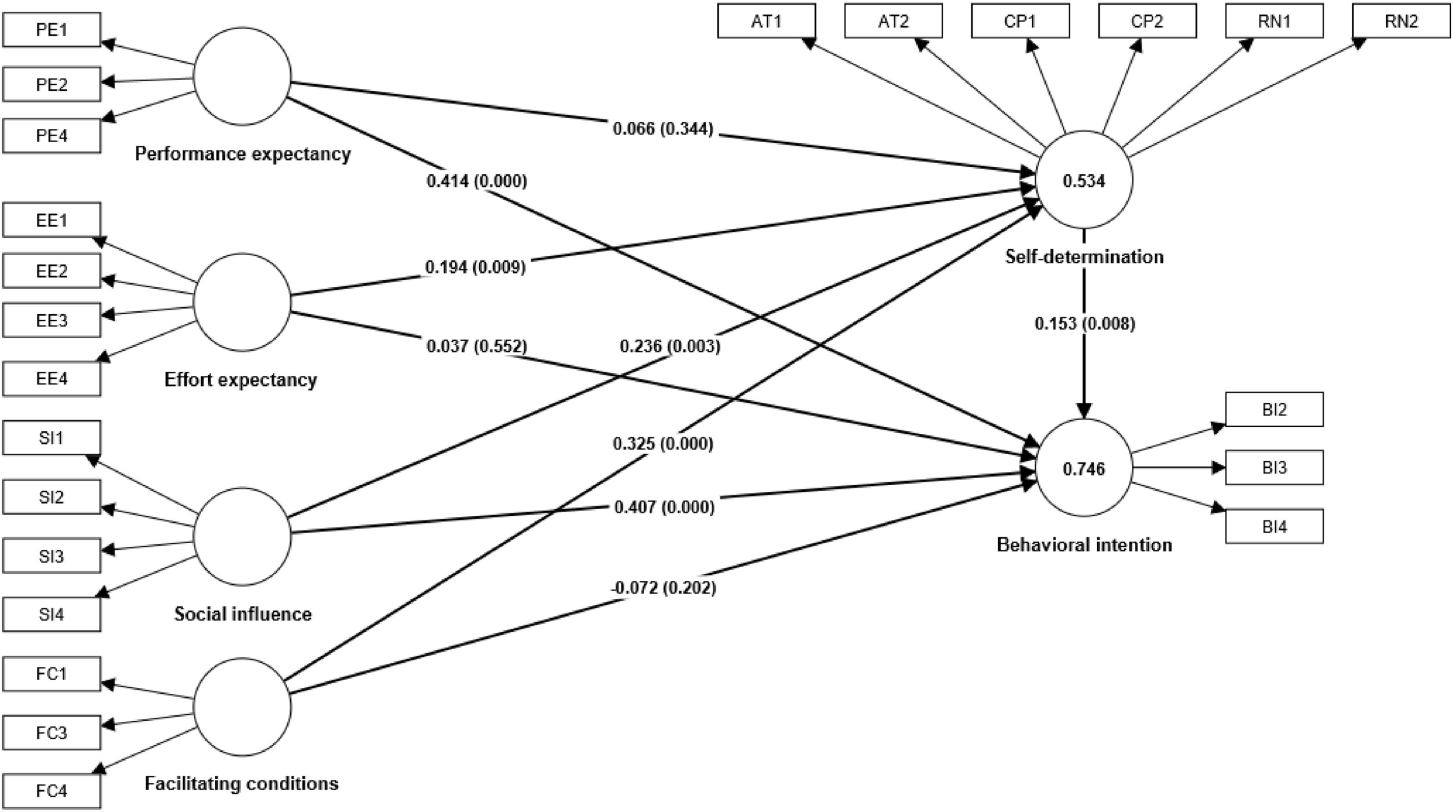
Final model with R^2^, path coefficients, and *p*-values. Values displayed as 0.000 in the SmartPLS output indicate *p* < 0.001.

A model’s capacity to explain variance is closely linked to its fit with the existing data, which is indicated by the strength of the relationships in the PLS path model. The coefficient of determination (R^2^) is the conventional measure used to evaluate the explanatory power of a structural model. It represents the proportion of variance in the endogenous variable that is accounted for by the connected exogenous variables (Hair et al., 2022, p. 195). The *R*^2^ values for self-determination and behavioral intention are 0.529 and 0.742, respectively, indicating that the model has considerable explanatory power, especially for behavioral intention. The f^2^ effect size measures the change in the *R*^2^ value when a specific antecedent construct is removed from the model. An *f*^2^ value between 0.02 and < 0.15 indicates a small effect, 0.15 to < 0.35 indicates a medium effect, and an *f*^2^ value of 0.35 or higher indicates a large effect (Hair et al., 2019). As shown in Table 9, performance expectancy (*f*^2^ = 0.228) and social influence (*f*^2^ = 0.174) both have a medium effect size on behavioral intention. Effort expectancy (*f*^2^ = 0.022), social influence (*f*^2^ = 0.033), and facilitating conditions (f^2^ = 0.095) have a small effect size on self-determination. The Q^2^ values for endogenous constructs were greater than zero, indicating that the model demonstrated satisfactory predictive relevance. The final model is displayed in Figure 2.

**TABLE 9:** Results of calculating f^2^ effect sizes.

Endogenous construct	Predictor construct				
	Performance expectancy	Effort expectancy	Social influence	Facilitating conditions	Self-determination
Self-determination (*R*^2^ = 0.534)	0.003	0.022	0.033	0.095	0.043
Behavioral intention (*R*^2^ = 0.746)	0.228	0.001	0.174	0.008	

## 5 Discussion

### 5.1 Discussion of findings

This study developed and tested an integrated UTAUT-SDT model to explain university students’ behavioral intentions to use education influencers. Overall, the findings suggest that both technology-related perceptions and motivational mechanisms play important roles in shaping students’ intentions in digital learning environments. In particular, the study highlights the importance of self-determination as an important motivational mechanism underlying behavioral intention.

In this study, self-determination was found to positively and significantly influence university students’ behavioral intentions to use education influencers. This finding indicates that students are more likely to engage with education influencers when they experience stronger feelings of autonomy, competence, and relatedness during the learning process. Compared with traditional classroom settings, education influencers often provide flexible, self-paced, and interest-driven learning experiences through social media platforms (Ahmed et al., 2024a). Such characteristics may strengthen students’ sense of autonomy because they can independently choose educational content according to their personal interests and learning needs. In addition, the interactive nature of social media platforms allows students to communicate with influencers and peers, which may enhance their sense of relatedness and social connection. Furthermore, simplified explanations, practical examples, and accessible learning content provided by education influencers may increase students’ perceptions of competence. Therefore, in the context of education influencers, motivational and psychological factors appear to play a particularly important role in shaping behavioral intention. Similar relationships between self-determination and behavioral intention have also been reported in previous educational technology studies (Alowayr and Al-Azawei, 2021; Bilquise et al., 2024; Osei et al., 2022).

Performance expectancy was found to have a significant direct effect on behavioral intention in this study, but it did not significantly influence self-determination. This finding suggests that university students may perceive education influencers as useful and beneficial for learning, even if these perceptions do not necessarily enhance their feelings of autonomy, competence, or relatedness. In the context of higher education, university students are often highly goal-oriented and may focus on whether educational content can improve academic efficiency, provide practical learning strategies, or support examination preparation. Therefore, when students believe that education influencers can enhance learning performance, they are more likely to develop intentions to use them. This finding is consistent with prior UTAUT-related studies showing that performance expectancy is an important predictor of behavioral intention (Chen et al., 2021; Liu et al., 2023). However, the non-significant relationship between performance expectancy and self-determination also suggests that perceived usefulness alone may not be sufficient to satisfy students’ deeper psychological and motivational needs.

Effort expectancy did not directly and significantly influence behavioral intention in this study, but it positively influenced self-determination. This finding may reflect the characteristics of contemporary university students, who are already highly familiar with social media platforms and digital technologies. Since education influencers mainly operate through commonly used platforms such as YouTube, TikTok, Instagram, and Bilibili, ease of use may already be regarded as a basic expectation rather than a decisive factor influencing behavioral intention. However, lower perceived effort may still contribute to students’ sense of competence and confidence in learning. When students perceive educational content as understandable, accessible, and easy to engage with, they may feel more capable of managing their own learning process. Although effort expectancy significantly predicted self-determination, its indirect effect on behavioral intention through self-determination was not statistically significant. This finding differs from some previous studies that identified a direct relationship between effort expectancy and behavioral intention (Zacharis and Nikolopoulou, 2022), suggesting that the role of effort expectancy may vary across different educational technology contexts.

Social influence significantly affected both self-determination and behavioral intention in this study. This result highlights the social nature of education influencer usage. Unlike many traditional educational technologies, education influencers operate within highly interactive social media environments in which peer recommendations, online communities, and social engagement are central components of the learning experience. University students may be influenced not only by the perceived educational value of influencers, but also by the behaviors and opinions of peers, classmates, and online communities. In addition, social interaction surrounding education influencers may strengthen students’ feelings of relatedness, which is one of the core dimensions of self-determination theory. Therefore, social influence appears to affect both students’ direct intention to use education influencers and their motivational engagement with learning. This finding is consistent with previous studies emphasizing the importance of social influence in educational technology adoption (Osei et al., 2022).

Facilitating conditions did not significantly influence behavioral intention directly in this study, although they significantly affected self-determination. One possible explanation is that university students already possess sufficient technological access and digital literacy to use education influencers. Because social media platforms are widely available and integrated into students’ daily lives, technical infrastructure and accessibility may no longer serve as major determinants of behavioral intention. However, facilitating conditions may still support students’ psychological needs indirectly. When students have stable access to digital devices, internet resources, and supportive learning environments, they may feel more capable of engaging with educational content independently, thereby enhancing their sense of competence and autonomy. Therefore, facilitating conditions may contribute to behavioral intention mainly through motivational pathways rather than through direct influence. Similar findings regarding the non-significant direct effect of facilitating conditions on behavioral intention have also been reported in previous studies (Foroughi et al., 2024).

Overall, the findings of this study suggest that integrating UTAUT and self-determination theory provides a more comprehensive explanation of university students’ behavioral intentions toward education influencers. While UTAUT explains how students evaluate the usefulness, ease of use, social influence, and environmental support associated with education influencers, SDT helps explain the underlying motivational and psychological mechanisms that connect these perceptions to behavioral intention. In particular, the results suggest that technology acceptance alone may not fully explain students’ intentions in social media-based learning environments. Instead, students’ feelings of autonomy, competence, and relatedness appear to play important motivational roles in shaping behavioral intention.

### 5.2 Theoretical and practical implications

Several implications can also be derived from the findings. Since self-determination significantly influenced behavioral intention in this study, educators and education influencers should consider creating learning environments that support students’ autonomy, competence, and relatedness. For example, education influencers may provide flexible learning pathways, interactive communication opportunities, and supportive online learning communities to strengthen students’ motivational engagement. In addition, because performance expectancy significantly influenced behavioral intention, educational content should be practical, relevant, and clearly connected to students’ academic needs. Education influencers may therefore improve students’ intentions to use educational content by providing concrete examples, effective learning strategies, and application-oriented explanations. Furthermore, the significant role of social influence suggests that collaborative learning communities and peer interaction may further strengthen students’ engagement with education influencers. Universities and educators may therefore consider integrating appropriate education influencer resources into higher education learning environments to support students’ digital learning experiences.

### 5.3 Limitations and future directions

This study also has several limitations. First, the participants were limited to university students in Nanjing, which may restrict the generalizability of the findings to other educational and cultural contexts. Second, the study adopted a cross-sectional research design, which limited the ability to examine changes in behavioral intention over time. Future studies may therefore employ longitudinal approaches to investigate how students’ motivations and intentions evolve in different stages of educational technology usage. Third, the study relied primarily on self-reported questionnaire data. Although this approach was appropriate for examining behavioral intention, future research could combine quantitative and qualitative methods, such as interviews or learning behavior analysis, to gain a more comprehensive understanding of university students’ experiences with education influencers.

## 6 Conclusion

This study investigated the factors influencing university students’ behavioral intentions to use education influencers by integrating the Unified Theory of Acceptance and Use of Technology (UTAUT) and Self-Determination Theory (SDT). Using PLS-SEM analysis based on 335 valid responses from university students, the study examined both the direct and indirect relationships among performance expectancy, effort expectancy, social influence, facilitating conditions, self-determination, and behavioral intention.

The findings provide clear responses to the research questions proposed in this study. Regarding RQ1, the results showed that performance expectancy, social influence, and self-determination significantly influenced university students’ behavioral intentions to use education influencers. In addition, effort expectancy, social influence, and facilitating conditions significantly influenced self-determination. However, effort expectancy and facilitating conditions did not directly affect behavioral intention. Regarding RQ2, the findings indicated that self-determination was a significant motivational predictor of behavioral intention, highlighting the importance of motivational and psychological mechanisms in university students’ adoption of education influencers.

The study also contributes theoretically by demonstrating the value of integrating UTAUT and SDT in the context of social media-based learning. While UTAUT explains students’ cognitive evaluations of education influencers, SDT helps explain the motivational processes underlying behavioral intention. The findings suggest that students’ feelings of autonomy, competence, and relatedness play an important role in connecting technology-related perceptions with behavioral intention. From a practical perspective, the findings suggest that educators and education influencers should not only focus on providing useful and accessible learning content, but also create supportive and interactive learning environments that strengthen students’ motivation and engagement. Universities may also consider incorporating appropriate education influencer resources into higher education learning environments to support students’ digital learning experiences.

Despite these contributions, this study has several limitations, including its cross-sectional design and the focus on university students from a single regional context. Future research may therefore explore longitudinal designs, cross-cultural comparisons, and mixed-method approaches to gain a more comprehensive understanding of students’ engagement with education influencers in higher education.

## Data Availability

The raw data supporting the conclusions of this article will be made available by the authors, without undue reservation.

## Footnotes

1
http://www.wjx.cn

## Appendix

Appendix 1 | Measurement items.

Performance Expectancy (PE)

PE1: Using education influencers improves my learning efficiency.

PE2: Education influencers help me better understand learning content.

PE4: Using education influencers enhances my academic performance.

Effort Expectancy (EE)

EE1: Learning through education influencers is easy for me.

EE2: It is easy for me to interact with education influencers.

EE3: I find education influencers easy to use for learning purposes.

EE4: It is easy for me to become skillful at using education influencers.

Social Influence (SI)

SI1: People who are important to me think that I should use education influencers.

SI2: My classmates or peers encourage me to use education influencers.

SI3: People around me support the use of education influencers.

SI4: Social media communities influence my use of education influencers.

Facilitating Conditions (FC)

FC1: I have the necessary resources to use education influencers.

FC3: I have access to the necessary technology to use education influencers.

FC4: I can get help when I encounter difficulties using education influencers.

Self-Determination (SD)

AT1: I use education influencers because I can freely choose learning content.

AT2: I feel autonomous when learning through education influencers.

CP1: I feel capable of learning effectively through education influencers.

CP2: Education influencers increase my confidence in learning.

RN1: I feel connected to others when using education influencers.

RN2: Education influencers help me feel part of a learning community.

Behavioral Intention (BI)

BI2: I intend to continue using education influencers for learning.

BI3: I will frequently use education influencers in the future.

BI4: I would recommend education influencers to others for learning purposes.
